# Endotoxin-Induced Monocytic Microparticles Have Contrasting Effects on Endothelial Inflammatory Responses

**DOI:** 10.1371/journal.pone.0091597

**Published:** 2014-03-19

**Authors:** Beryl Wen, Valery Combes, Amandine Bonhoure, Babette B. Weksler, Pierre-Olivier Couraud, Georges E. R. Grau

**Affiliations:** 1 Vascular Immunology Unit, Sydney Medical School & Bosch Institute, University of Sydney, Camperdown, Australia; 2 Weill Medical College, Cornell University, New York, New York, United States of America; 3 Institut Cochin, INSERM U1016, Paris, France; 4 CNRS, UMR 8104, Paris, France; 5 Université Paris Descartes, Sorbonne Paris Cité, Paris, France; Biological Research Centre of the Hungarian Academy of Sciences, Hungary

## Abstract

Septic shock is a severe disease state characterised by the body's life threatening response to infection. Complex interactions between endothelial cells and circulating monocytes are responsible for microvasculature dysfunction contributing to the pathogenesis of this syndrome. Here, we intended to determine whether microparticles derived from activated monocytes contribute towards inflammatory processes and notably vascular permeability. We found that endotoxin stimulation of human monocytes enhances the release of microparticles of varying phenotypes and mRNA contents. Elevated numbers of LPS-induced monocytic microparticles (mMP) expressed CD54 and contained higher levels of transcripts for pro-inflammatory cytokines such as TNF, IL-6 and IL-8. Using a prothrombin time assay, a greater reduction in plasma coagulation time was observed with LPS-induced mMP than with non-stimulated mMP. Co-incubation of mMP with the human brain endothelial cell line hCMEC/D3 triggered their time-dependent uptake and significantly enhanced endothelial microparticle release. Unexpectedly, mMP also modified signalling pathways by diminishing pSrc (tyr416) expression and promoted endothelial monolayer tightness, as demonstrated by endothelial impedance and permeability assays. Altogether, these data strongly suggest that LPS-induced mMP have contrasting effects on the intercellular communication network and display a dual potential: enhanced pro-inflammatory and procoagulant properties, together with protective function of the endothelium.

## Introduction

Microparticles (MP) are a population of small vesicles derived from host cell plasma membranes, ranging between 0.2–1 µm in diameter. First described by Wolf in 1967 as ‘platelet dust’ [1], these seemingly inert vesicles are present in the circulation of normal healthy subjects and have since been proposed as regulators of vascular homeostasis under physiological conditions [2]. Their enhanced release is triggered by cell injury, activation or apoptosis and various clinical studies have shown an association between MP levels and disease severity [3]–[6].

The MP formation process, named vesiculation, is complex and yet to be fully deciphered, with different agonists capable of inducing different MP profiles. However, it is accepted that MP bear a negatively charged outer leaflet with exposed phosphatidylethanolamine and phosphatidylserine (PS), and a positively charged inner membrane leaflet where phosphatidylcholine and sphingomyelin almost exclusively reside [7], [8]. Being released from a range of different cell types, MP display phenotypic and cytosolic compositions that tend to mirror those of their mother cell. This could account for their active, procoagulant and inflammatory nature often observed in vascular functional studies [9]–[12].

Increased levels of circulating MP have been measured in many disease states and are closely associated with disease severity. For example, increased levels of MP derived from monocytes were found in patients with cancer, diabetes and hypertension [3], [13] compared to healthy individuals. Acting as intermediate messengers, monocytic MP (mMP) are able to transfer biologically active molecules such as IL-1β and caspase-1 to target cells, subsequently altering the functional capacity of the latter [14], [15]. mMP are capable of inducing endothelial oxidative stress and upregulating tissue factor and von Willebrand factor expression to trigger downstream thrombotic events [16]. Additionally, recent studies have reported that mMP are capable of inducing endothelial nitrosative stress [17]. Whilst many studies implicate a deleterious role for mMP, the actual mechanism describing such a role remains to be confirmed.

The elevated level of mMP in infectious diseases such as sepsis is well established yet their participation in the pathophysiology of sepsis is still being investigated [18], [19]. One of the most important nosocomial diseases, sepsis encompasses a diverse array of pathological sequelae leading to a death rate of up to 70% in the USA and 30% in Australia [20], [21]. This severe disease state is attributed more to the dysregulated inflammatory response to infection than to the infection itself. One of the major neurological complications is septic encephalopathy, which, in close association with mortality, can occur in 8 to 71% of patients with sepsis [22]–[24]. Despite extensive research in the area of sepsis, severe sepsis and septic shock, the pathophysiological mechanisms of this disease state remains poorly understood as evinced by continued new strategies proposed for sepsis treatment [25], [26].

Under normal conditions, the blood vessels have an important role in maintaining homeostasis by regulating inflammatory mediators and controlling responses such as vascular tone modulation and thrombus formation. During sepsis, the endothelium –including that of the blood brain barrier - can undergo changes in blood flow, permeability and leukocyte trafficking in an attempt to maintain homeostasis (reviewed in [27]). Under inflammatory conditions, disturbances to the blood-brain barrier can alter the conformation of tight junctions leading to a functionally compromised barrier. Such modifications affecting monolayer integrity and thus changes in endothelial permeability lead to the influx of cells, proteins and excess fluids as is observed in sepsis [28]. The subsequent formation of oedema has the potential to further compromise microvascular viability and tissue perfusion, exacerbating the severity of the disease [29]. In addition to vascular changes during inflammation, circulating blood cells undergo a series of responses to inflammatory stimuli. Large numbers of circulating activated monocytes and their ability to traverse the blood-brain barrier contribute to the pathogenesis of this disease [30]. Multiple studies indicate that the excessive release from monocytes and macrophages of pro-inflammatory cytokines such as IL-1β, IL-6 and TNF [31] is an important propagating factor in septic shock and may contribute to multiple organ failure [32]. As monocytes can trigger the inflammatory response, MP released by them could also participate in the pathogenesis of septic shock. The pathophysiological role of monocytes has long been linked with inflammation, especially through alteration of the endothelial monolayer although their exact involvement or that of mMP in the disease process has not been clarified. Additionally, there is limited information on whether their MP progeny may serve as intermediate mediators of cell-cell communication and amplify the endothelial cell response to monocytic activation.

Therefore, we compared mMP with monocytes for contributions to inflammatory processes. We hypothesised that mMP generated by endotoxin stimulation of monocytes could directly elicit significant endothelial changes. In particular, we evaluated whether mMP disrupted the blood brain barrier and investigated the subsequent downstream events.

## Materials and Methods

### Reagents

TNF was obtained from Peprotech (London, UK), LPS (from *Escherichia coli* O111:B4) and cytochalasin D from Sigma (Saint Louis, MO, USA). The following monoclonal antibodies for flow cytometry were obtained from Beckman Coulter Immunotech (Marseille, France): anti- human CD54, CD11b and CD14. Antibodies to HLA-DR were from eBioscience, to CD31 and those to tissue factor from BD Pharmingen (San Diego, CA, USA). Annexin V-FITC was from Beckman Coulter. Rabbit anti-pSrc-family (Tyr416) and mouse anti-Src antibodies were from Cell Signalling Technologies; mouse-anti-GAPDH (clone 6C5) antibody was from Millipore; rabbit anti-ZO-1 antibody was from Invitrogen and rabbit anti-VE-Cadherin was from Sigma (Saint Louis, MO, USA). Secondary anti-rabbit IgG conjugated to DyLight 800 and anti-mouse IgG conjugated to Dylight 680 were from Cell Signalling Technologies. For microscopy goat-anti-rabbit Alexa-Fluor 546 IgG and Pro-long mounting medium containing DAPI were from Invitrogen.

### Cell culture

The immortalised monocytic cell line Mono Mac-6 (MM6), a human cell line with characteristics of mature monocytes, was a kind gift from Ziegler-Heitbrock [33] and the monoblastic cell line THP1 was a kind gift from Saunders [34]. Both monocytic cell lines were maintained in RPMI medium (Invitrogen) supplemented with 10% heat inactivated foetal calf serum (FCS) (Bovogen) at 37°C in 5% CO_2_.

The human brain microvascular endothelial cell line hCMEC/D3 [35] was cultured in endothelial cell basal medium-2 (Lonza) supplemented with 5% FCS, recombinant long R insulin-like growth factor-1 (R-IGF-1), vascular endothelial growth factor, ascorbic acid, hydrocortisone, epidermal growth factor human recombinant and human fibroblast growth factor-B (all from Lonza). The cells were seeded onto 0.3% collagen coated flasks and grown at 37°C in 5% CO_2_.

### MP production

Endothelial cells were seeded onto a 0.3% collagen coated 24-well plate at 6×10^4^ cells/ml and grown for 48 hours until 80% confluence was reached. To better mimic inflammatory conditions during endotoxic shock, endothelial cells were then stimulated with TNF (0.2 to 100 ng/ml) for 18 hours.

MM6 and THP1 monocytes were washed and resuspended in fresh RPMI culture medium, counted and seeded onto a 6-well plate (1×10^6^ cells/ml) and treated with LPS at100 ng/ml for 18 hours at 37°C. Cell viability was assessed by trypan blue assay.

To harvest either endothelial MP (eMP) or monocytic MP (mMP), supernatant medium from each cultured cell line was centrifuged at 500 g for 5 minutes at 25°C to pellet cells and the resulting supernatant was re-centrifuged at 1,200 g for 5 minutes to remove cell debris. The final MP pellet was obtained after two further centrifugations at 18,000 g for 45 minutes at 16°C conducted with washes in between. MP purity was assessed by flow cytometry.

eMP were labelled with anti-CD105-PE and mMP were stained with either anti-CD31-FITC or annexin-V-FITC for 45 minutes in 10× binding buffer and enumerated by flow cytometry on the Beckman Coulter FC500 using Flow-Count™ fluorospheres as an internal standard (Beckman Coulter). The MP region was defined using a FSC-SSC dot plot as previously described [36]. Briefly, the upper MP region was set using 0.8–1.1 µm latex beads and the number of fluorescent of events lying within this MP gate was measured.

### Endotoxin detection in MP preparations

Purified MP were analysed for the presence of endotoxin using a Limulus Amebocyte Lysate Endotoxin Assay Kit purchased from Lonza. Samples were run according to manufacturer's instructions.

### Phenotyping of monocytes and their derived MP

After LPS stimulation, MM6 and THP1 monocytes were counted, washed and labelled for CD54, CD11b, HLA-DR, CD14, CD31 and tissue factor expression according to manufacturer's instructions. Isotype-matched controls were used for each antibody. Following incubation, excess unbound antibodies were washed away and the cells were suspended in RPMI for flow cytometry analysis. Mean fluorescence intensity and percentage of cells positive for each marker were compared to the values obtained in resting, unstimulated monocytes.

After purification, equal numbers of mMP derived from resting and LPS-stimulated cells were incubated with 3 µL of antibody against CD54, CD11b, HLA-DR, CD14, CD31 or tissue factor for 45 minutes at room temperature in the dark. Isotype-matched control antibodies were also used. Samples were subjected to flow cytometry (Beckman Coulter FC500) and results analysed using CXP software.

### Characterisation of monocytes and mMP cytosolic content

Total RNA from each monocyte or mMP preparation was extracted using the RNeasy Mini Kit (Qiagen) instructions followed by incubation with random primers (10% v/v) (Fermentas) at 70°C for 10 minutes. Reverse transcription was performed using a M-MLV Reverse Transcriptase RNase H- kit (Solis BioDyne) according the manufacturer's instructions with the addition of dNTP (1 mM) (Fermentas) and RNase OUT (2.5% v/v) (Invitrogen) followed by enzyme heat inactivation for 5 minutes at 92°C. The resulting synthesised cDNA were probed with Sensimix (Quantace) and the following RT-PCR primers: RPL13A (sense: 5′-CGCCCTACGACAAGAAAAAG, antisense: 5′-CCGTAGCCTCATGAGCTGTT), CD80 (sense: 5′-GGACATGAATATATGGCCCG, antisense: 5′-CAACACACTCGTATGTGCCC), CD86 (sense: 5′-ACAGCAGAAGCAGCCAAAAT, antisense: 5′-CTTGTGCGGCCCATATACTT), tissue factor (sense: 5′- CCGAACAGTTAACCGGAAGA, antisense: 5′ -CTTCACAATCTCGTCGGTGA), ICAM-1 (sense: 5′-CTCCTCTGCTACTCAGAGTT, antisense: 5′-CAT ACACCTTCCGGTTGTTC), VCAM-1, (sense: 5′-ATGCCTGGGAAGATGGTCG, antisense: 5′-TCTGGGGTGGTCTCGATTTTA), IL-6 (sense: 5′-TGTAACCATGGACCCAATATTTACC, antisense: 5′-AAGACAGTAACAGCTTAAACCTGGAAA), IL-8 (sense: 5′-GGAATTGAATGGGTTTGCTAGAAT, antisense: 5′-TGTGGATCCTGGCTAGCAGACT), TLR-4 (sense: 5′-CCAGGATGAGGACTGGGTAA, antisense: 5′- TACCAGCACGACTGCTCAG), TNF (sense: 5′- AGGGCCTGTACCTCATCTACTCCC, antisense: 5′-ACCTTGGTCTGGTAGGAGACGGC), HLA-DR (sense: 5′-CCGAGGATTTCGTGTACCAG, antisense: 5′- GCACGACCTCTTCTCGGTTA).

The samples were amplified with 40 cycles of denaturation at 94°C for 60 seconds, annealing at 54°C for 45 seconds followed by an extension at 72°C for 2 minutes using a Rotorgene PCR machine (Corbett Research).

### Clot time measurement

Control normal pooled plasma (Stago Diagnostica, #0678) and neoplastin (Stago Diagnostica, #0665) were solubilised according to manufacturer's instructions. MP derived from non-stimulated and LPS-treated monocytes were counted and equal numbers were added to control plasma before being loaded onto the semi-automated #STart4 coagulometer (Stago Diagnostica). After 30 seconds of incubation at 37°C, an equal volume of neoplastin was added and the clot formation time was measured.

### Co-culture of mMP with human brain endothelial cells

MP derived from either resting or LPS-stimulated MM6 and THP1 were co-incubated with a confluent monolayer of either resting or TNF-primed endothelial cells (0.2 ng/ml) at a ratio of 10 mMP: 1 endothelial cell for 18 hours at 37°C for all conditions unless otherwise stated. mMP supernatants from final ultracentrifugations were used as an additional control. eMP were stained with anti-CD105-PE mAb and quantitatively analysed by flow cytometry as previously described.

### Protein analysis of human brain endothelial cells

Western blots were performed to observe changes in endothelial protein expression resulting from incubation with mMP. Briefly, after denaturation in lysis buffer, endothelial cell lysates were separated on an 8% polyacrylamide gel by electrophoresis and transfer blotted onto nitrocellulose membrane (Amersham). Membranes were incubated in Odyssey blocking buffer for 1 hour at room temperature before being probed for pSrc-family (Tyr 416), Src, ZO-1, VE-cadherin and GAPDH overnight at 4°C. The membranes were then washed and incubated with fluorescently conjugated secondary antibodies for 1 hour at room temperature. Fluorescent protein expression was analysed using the Odyssey Imaging System (LICOR). pSrc was expressed as the relative fluorescence after normalisation to Src total. ZO-1 and VE-cadherin were both normalised against GAPDH.

### Impedance studies

Electrode arrays (Applied BioPhysics, #8W1E) were pre-treated with L-cysteine (10 mM) (Sigma) for 15 minutes, washed twice in sterile water and then coated with 0.3% collagen for 1 hour. Endothelial cells were seeded at 1.5×10^5^ cells/ml and loaded into the electrical cell-substrate impedance sensing (ECIS) morphological biosensor (Applied Biophysics) at 37°C for a minimum of 48 hours. Once confluence was attained, endothelial cells were incubated with TNF (0.2 ng/ml) at 37°C for 18 hours. Equal numbers of mMP purified from resting or LPS (100 ng/ml) treated monocytes were added to each endothelial cell condition and loaded onto the ECIS. Impedance readings of the endothelial monolayer were taken at 10 minute intervals for 48 hours.

### Measurement of 70 kDa Dextran Permeability

Endothelial cells were seeded onto 0.4 µm pore size, collagen coated Transwell inserts in 24 well plates at 3×10^4^ cells/ml and grown until confluent. The experimental inserts were stimulated with a low dose of TNF (0.2 ng/ml) overnight at 37°C. MP purified from resting or LPS-treated monocytes were then added and co-cultured with the endothelial cells for 24 hours at 37°C. Overnight treatments of hCMEC/D3 with TNF (100 ng/ml) or 1 hour treatments with cytochalasin-D (10 µg/ml) were used as positive controls for loss of monolayer integrity.

Endothelial culture medium was replaced with 70 kDa FITC-dextran (1 mg/ml) (Invitrogen) diluted in DMEM without phenol red (Gibco) in the upper chamber. After gentle resuspension of the lower chamber, 50 µl samples were removed at times = 0, 45, 90, 150 and 240 minutes and the fluorescence intensity measured on a Fluostar Optima (BMG Labtech).

### Visualisation of endothelial junctions

Purified MP from resting and LPS-stimulated monocytes were co-cultured with confluent resting or TNF-pre-stimulated endothelial cells at a ratio of 10 mMP:1 endothelial cell overnight at 37°C. Unbound mMP were then removed and endothelial cells were washed in PBS before fixation with 1% paraformaldehyde for 30 minutes at room temperature. Cells were then permeabilised in 0.1% Triton X-100/PBS for 5 minutes, washed and blocked with 2% BSA/0.1% Triton-X-100/PBS for 1 hour. Samples were incubated with primary antibodies α-VE-cadherin or α-ZO-1(1∶200 dilution in blocking buffer) for 40 minutes, washed and fluorescently labelled with Alexa Fluor-546 (1∶800 dilution in blocking buffer) overnight at 4°C. Cells were then washed and mounted in Pro-long gold antifade reagent containing DAPI. Samples were viewed using the Olympus IX71 deconvolution fluorescence microscope for wide field images and the Zeiss LSM 510 Meta Spectral Confocal microscope.

### Statistics

Results are shown as mean ± S.D and were analysed using GraphPad Prism 5 software. For statistical analyses, the one way ANOVA followed by the Tukey post-test and the Kruskall Wallis followed by the Dunn's test were used. Comparative statistical analyses between two groups were performed using the Mann Whitney test.

## Results

### Enhancement of endothelial and monocytic MP production by TNF and LPS

**Figure 1:**
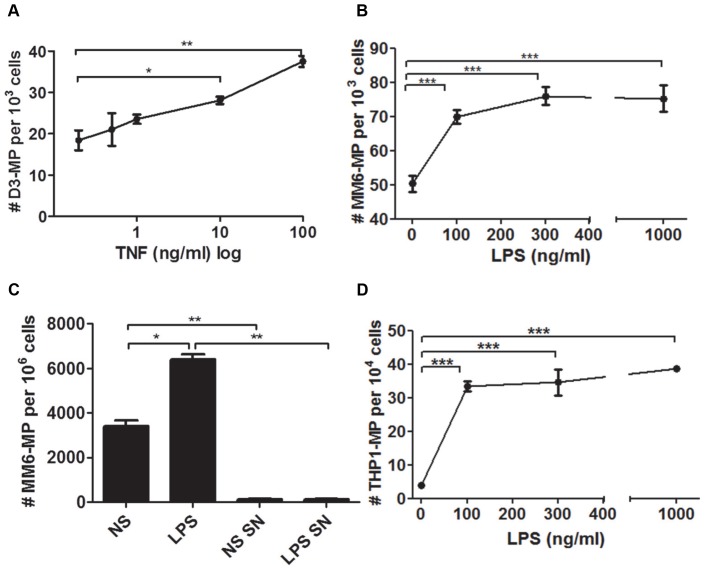
Determining optimal agonist concentrations on hCMEC/D3 and monocytes. Endothelial cells and monocytes were stimulated overnight with varying doses of TNF and LPS, respectively. Induced eMP were measured by flow cytometry using PE-anti-CD105 mAb (A). MP released from monocytes were counted directly from the cell suspension prior to any purification process using labelling with FITC-anti-CD31 mAb (B). Post purification, MP from non-stimulated (NS) and LPS-stimulated monocytes were enumerated and the supernatant (SN) from the final centrifugation process was also checked to ensure clearance of mMP (C). Experiments were performed at least 3 times in triplicates. Data are expressed as mean ± SD. *p<0.05, **p<0.01 ***p<0.001.

Overnight stimulation of endothelial cells with TNF resulted in a dose-dependent increase in the number of eMP released (Figure 1A). These endothelial cells shed 18001±1645 MP under resting conditions while stimulation with TNF at 10 ng/ml significantly increased endothelial vesiculation. Maximal levels of eMP were induced by TNF 100 ng/ml. In contrast, TNF 0.2 ng/ml did not significantly modify eMP release levels from basal levels but was sufficient to upregulate adhesion molecules such as ICAM-1 and VCAM-1 (personal data and [37], [38]). Thus this sub-optimal concentration was chosen to prime the endothelial cells without inducing significant eMP release.

LPS treatment of both monocytic cell lines MM6 and THP1 enhanced the release of mMP as detected using anti-CD31 mAb (Figure 1B, 1D). As all three doses elicited a significant response from the monocytes, the dose of LPS 100 ng/ml was chosen as a sufficient concentration for mMP release without significantly compromising cell viability, as assessed by the trypan blue exclusion method (data not shown). After MP purification, a two-fold increase of basal vesiculation was still observed after LPS stimulation and the final supernatant was mostly free of MP (Figure 1C). Similarly, LPS treatment of the monocytic cell line THP1 significantly enhanced mMP release at all three doses tested (Figure 1D). The dose of LPS 100 ng/ml induced up to a six-fold increase of basal vesiculation and was chosen as the optimal dose for mMP release without significantly compromising cell viability.

### Surface and cytosolic content profiling of monocytes and mMP

Endotoxin levels were measured in MP samples purified from LPS-treated monocytes using a Limulus amebocyte lysate endotoxin assay. The level of endotoxin in MP purified from both resting and LPS-stimulated monocytes were below the detectable threshold. We also tested several concentrations of LPS between 1 ng/ml and 1 µg/ml, of which the concentration used for monocytic activation (100 ng/ml) was significantly higher than the maximum standard provided (1 EU).

**Figure 2:**
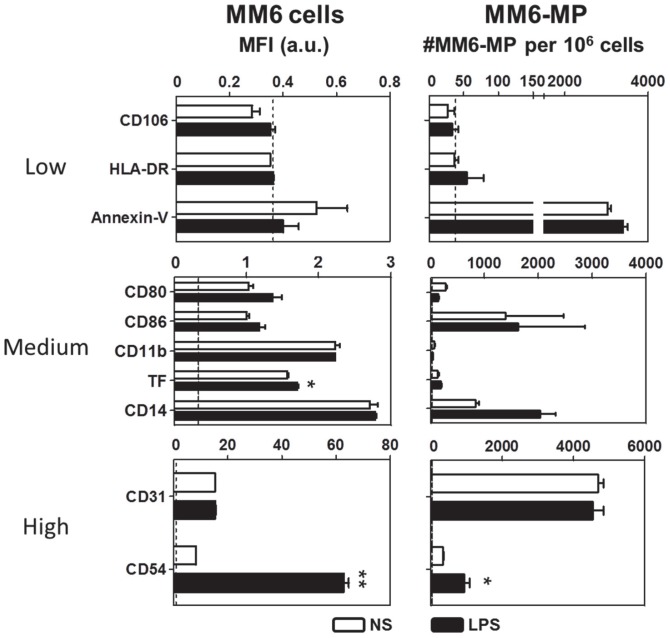
Monocyte and mMP surface antigen phenotype. Both unstimulated and LPS-stimulated MM6 were stained with anti-CD106, HLA-DR, CD80, CD86, CD11b, TF, CD14, CD31, CD54 mAb and annexin-V. The mean fluorescence intensity was measured and compared to isotype-matched controls (left column). mMP were also stained for the same surface antigens to check inheritance from the parent cell. Positively stained MP were counted and expressed as the number of MP per 10^6^ monocytes (right column). Monocytes with MFI between 0–1, 1–5, and above 10 were considered as low expressors (top panel), medium expressors (middle panel) and high expressors (bottom panel) respectively. Experiments were performed at least 3 times in duplicates. Data are expressed as mean ± SD. *p<0.05, **p<0.01.

After overnight treatment with LPS, MM6 and their derived MP were characterised for surface antigen expression by flow cytometry (Figure 2). We observed that similarly to monocytes, mMP expressed low or non-detectable levels of HLA-DR or CD106 (VCAM-1, used as a negative control), respectively, whether or not they were stimulated with LPS. In contrast, although monocytes had low surface protein expression of PS indicated by annexin-V binding, up to 45% of mMP were PS-positive by annexin V labelling. Compared to the surface antigens tested, monocytes expressed intermediate levels of CD11b, tissue factor (TF) and CD14. LPS treatment did not significantly upregulate these antigens, although a higher percentage of LPS stimulated cells were positive for them compared to unstimulated monocytes (35% vs. 42% for CD11b, 56% vs. 74%, for TF, 36% vs. 40% for CD14, respectively for unstimulated vs. LPS treated monocytes, data not shown). CD80 and CD86 were detected on 16% and 46% of resting monocytes, respectively. Despite the increased percentage of monocytes expressing CD80 (27.48%) and CD86 (69%), quantitative expression levels were not modulated by LPS stimulation. Virtually all (98.33±0.19%) monocytes exhibited high CD31 expression as did their MP progeny. Interestingly, there was no significant change in the numbers of MP positive for CD80 and CD86 between MP harvested from resting monocytes or from LPS stimulated monocytes. The number of CD31-postitive mMP also did not change with LPS stimulation. LPS was, however, able to increase the percentage of CD54-positive cells from 86% to 97%, and to significantly upregulate monocytic expression of CD54. Moreover, higher numbers of mMP were CD54-positive when derived from LPS stimulated cells. Similar results were observed in the more monoblastic THP1 cell line (see Fig. S1).

**Figure 3:**
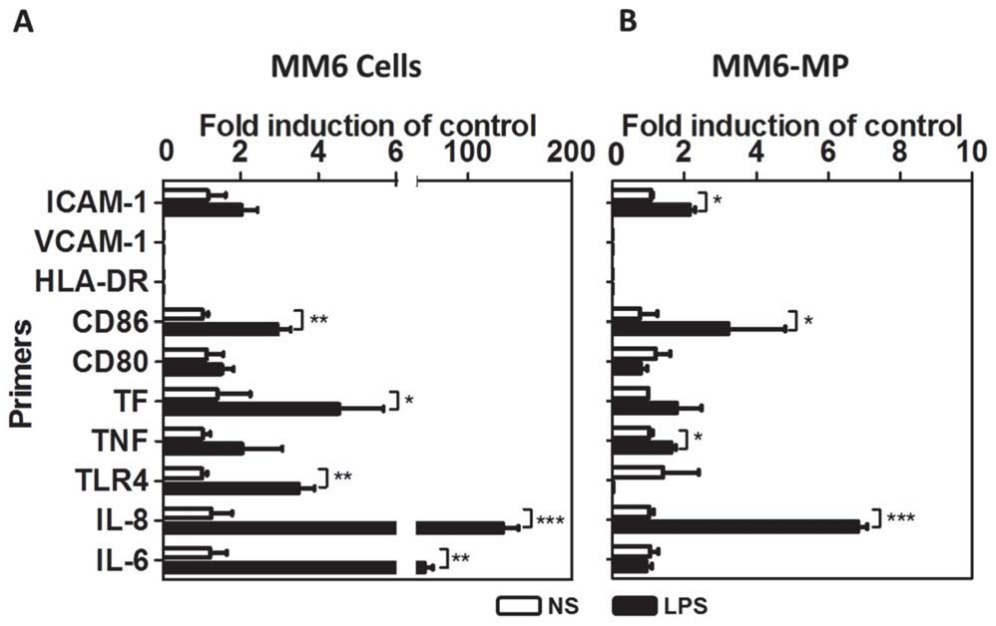
Cytosolic mRNA profiling of monocytes and mMP. RNA was extracted from resting (open bars) or stimulated (black bars) monocytes (A) and their induced MP (B) and the sequences were amplified using RT-qPCR. Results were taken as the level relative to expression at resting levels. Experiments were performed at least 3 times in duplicates. Data are expressed as mean ± SD. *p<0.05, **p<0.01, ***p<0.001.

The modulation of transcripts for cytokines and surface molecules upon LPS stimulation was then tested in MM6 and their derived MP (Figure 3). Amplification of IL-6, IL-8, TLR4, TF and CD86 mRNA from MM6 cells revealed a significant accumulation of these mRNAs 18 hours after LPS. mRNAs from TNF, CD80 and ICAM-1 were detected but no significant increase in LPS-stimulated cells was observed. The cells did not contain detectable levels of HLA-DR or VCAM-1, the latter taken as a negative control (Figure 3A).

Similarly, in MM6- derived mMP, amplification of IL-8, TNF, CD86 and ICAM-1 mRNA revealed significantly increased levels of the mRNA sequences upon LPS stimulation of the monocytes. No significant change was observed between non-stimulated and LPS-stimulated mMP for IL-6, TF and CD80. TLR4 mRNA expression appeared to be down regulated in LPS induced mMP. These mMP also did not express any sign of HLA-DR or VCAM-1 (Figure 3B).

### mMP are procoagulant

**Figure 4:**
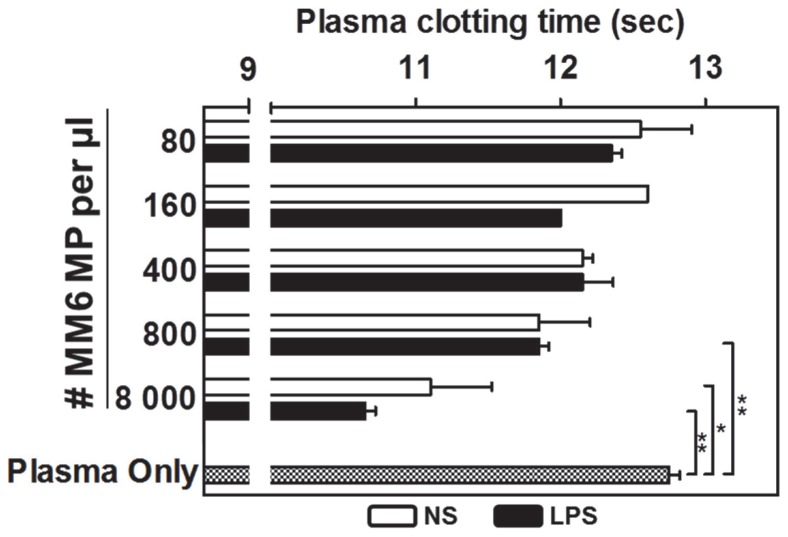
Procoagulant potential of mMP. mMP from resting and LPS-stimulated cells were added to normal plasma pool and the change in clotting time measured. Low doses of mMP did not induce any changes in clotting time. However, higher numbers of mMP induced a significant reduction in plasma clotting time. LPS-induced MP (black bars) also appeared to be more procoagulant than MP from resting monocytes (open bars). Results are representative of 3 independent experiments performed in duplicates. Data are mean ± SD. *p<0.05, **p<0.01.

MP purified from resting and LPS-stimulated monocytes were subjected to a prothrombin time assay. Under standard conditions, normal pooled plasma clotted in 12.7±0.05 seconds (Figure 4). The addition of mMP reduced clotting time in a concentration-dependent manner. More notably, fewer LPS-induced mMP were needed for a significant reduction in clotting time - 800 MP/µl (p<0.01) - with further shortening when 8000 MP/µl were added to the assay system (p<0.01). In contrast, more MP from non-stimulated monocytes were needed i.e. 8000 MP/µl, before a significant reduction in clotting time was observed. (p<0.05). At low MP numbers, i.e. 400 MP/µl, no significant difference was observed between the procoagulant potential of non-stimulated and LPS-induced mMP.

### mMP bind to endothelial cells and induce their vesiculation

**Figure 5:**
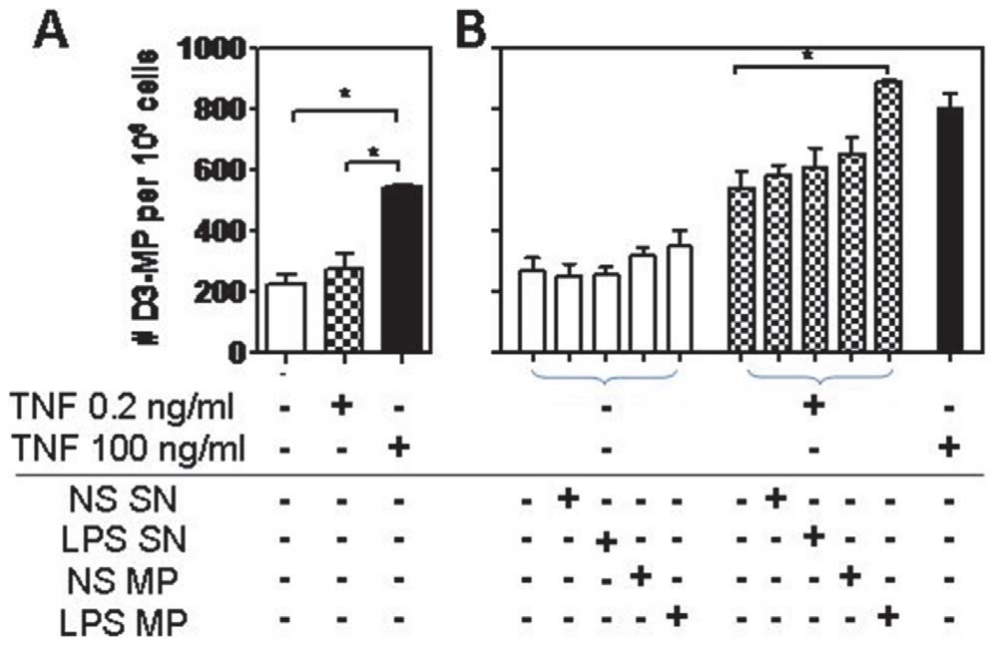
Interaction and effect of mMP on eMP vesiculation. Endothelial cells were primed or activated with TNF overnight and the levels of MP released were checked before co-incubation with mMP to ensure cells were optimally responsive (A). After co-incubation with mMP, controls levels of eMP rose cumulatively (B). Non-stimulated mMP and final SN did not induce any significant changes. Data represents duplicates of 4 independent experiments. Data are expressed as mean ± SD. *p<0.05.

Supernatant media from unstimulated, TNF-primed or TNF-stimulated endothelial cell monolayers were analysed for eMP release to define the baseline vesiculation levels prior to co-incubation with mMP. As expected, stimulation of cells with TNF 100 ng/ml significantly increased the numbers of eMP released while no change was observed in TNF-primed cells (Figure 5A).

Following an overnight incubation with MM6 mMP, the number of eMP from non-stimulated, TNF-primed and TNF-stimulated endothelial cells cumulatively rose (Figure 5B). Although adding mMP to resting endothelial cells produced no change in eMP release compared with untreated endothelial cells, the co-incubation of mMP with TNF-primed endothelial cells enhanced the number of eMP shed in comparison to release after TNF priming alone. As a control, supernatant media from both non-stimulated and LPS-stimulated mMP final purifications did not significantly change the number of eMP released compared to the medium alone. Stimulation of endothelial cells with mMP from THP1 revealed similar results whereby enhanced eMP release was observed in all conditions, and was significantly enhanced when endothelial cells were TNF-primed and treated with mMP derived from LPS treated THP1. (See Fig. S2).

### Effect of mMP on brain endothelium integrity

**Figure 6:**
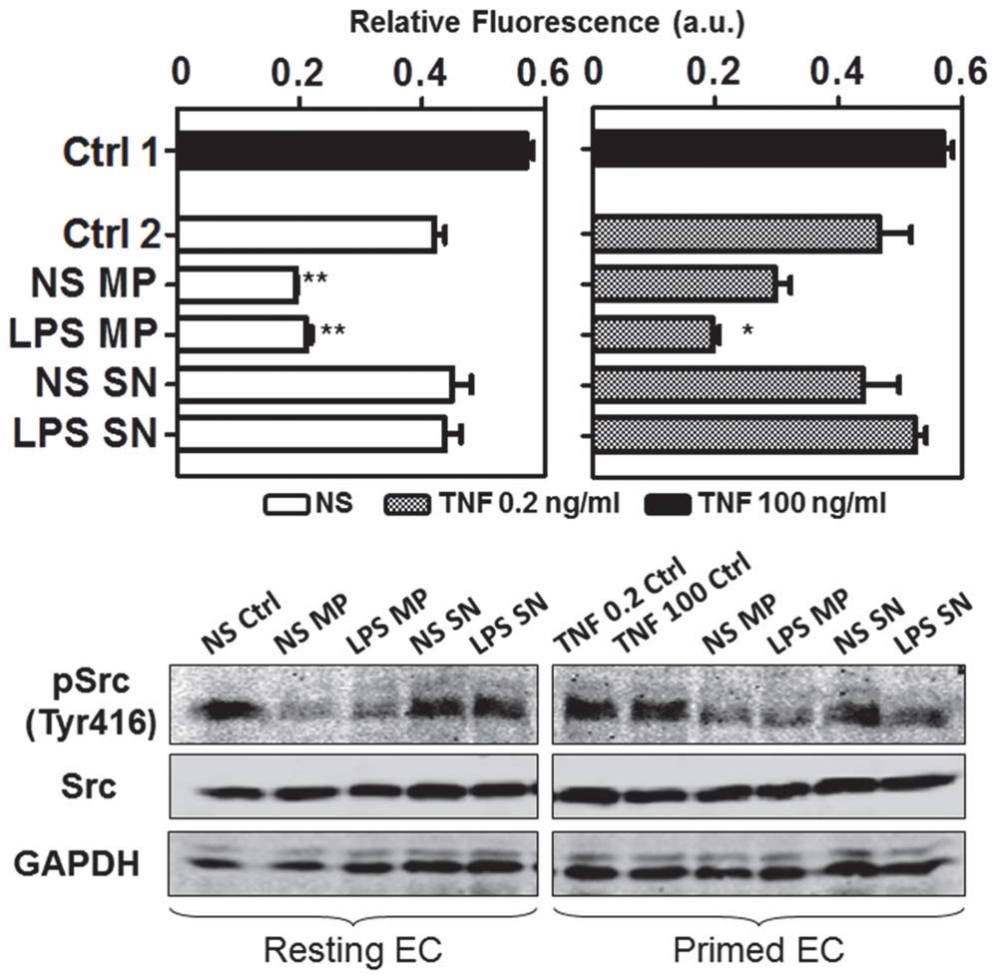
mMP induced protein expression in endothelial cells. Endothelial protein expression of pSrc (Tyr416) and Src were examined after treatment with mMP. GAPDH was used as a loading control. Non-stimulated mMP had a more pronounced effect on resting (top left) rather than TNF-primed endothelial cells (top right). LPS mMP significantly decreased pSrc expression in both resting and TNF primed endothelial cells. Neither SN from the final NS or LPS induced mMP pellet had any effect. Non-stimulated (NS), TNF primed (TNF 0.2 ng/ml) and activated (TNF 100 ng/ml) endothelial cells are represented by open, grey and black bars respectively. Actual protein expression of pSrc, Src and GAPDH are shown in lower panels. Data represent three independent experiments. Data are mean ± SD. *p<0.05, **p<0.01.

Protein content of endothelial cells was examined to determine whether mMP influenced certain endothelial translational or phosphorylation events. The cytosolic tyrosine kinase Src is known to be involved in multiple signalling pathways, including control of endothelial permeability. Therefore we probed for its activated and phosphorylated form in our endothelial cells. Exposure of endothelial cells to TNF at 0.2 ng/ml increased pSrc expression by 20% while TNF at 100 ng/ml increased pSrc expression by 35%. Co-incubation of endothelial cells with mMP resulted in a diminished expression of pSrc protein in both the resting and TNF primed conditions (Figure 6). It was also observed that under resting conditions, non-stimulated mMP limited pSrc expression more than did LPS-induced mMP; the reverse was true for TNF-primed endothelial cells.

**Figure 7:**
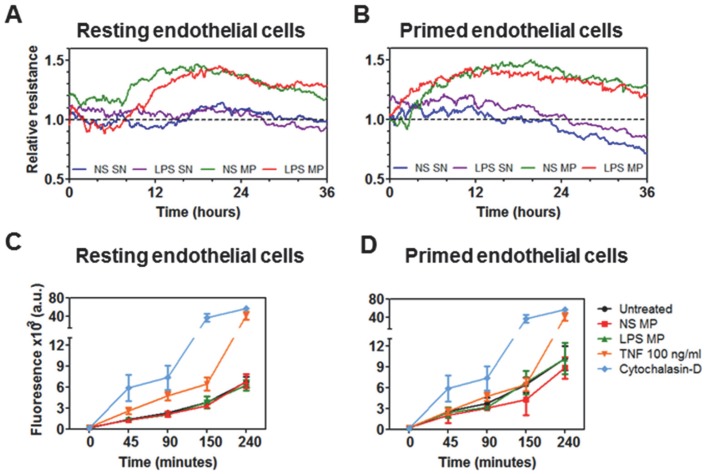
Effect of mMP on endothelial cell monolayer. Co-incubation of mMP with resting endothelial cells induced an increase in monolayer impedance (A). Similarly, co-culturing mMP onto pre-stimulated endothelial cells induced raised impedance of the endothelial monolayer whereas stimulation with TNF alone (or with SN) decreased TEER (B). The SN did not have any effect. After overnight co-culture, mMP did not alter the passage of FITC-dextran through either resting (C) or TNF-primed endothelial monolayers (D) over 4 hours. Data shown are representative of three independent experiments. FITC-dextran permeability assays were performed in triplicates and expressed as means ± SD.

After endothelial cells reached confluence, the impedance of the unstimulated endothelial monolayer remained constant for 36 hours (Figure 7A). Upon the addition of mMP (whether or not LPS-stimulated), the impedance of the monolayer began increasing after approximately 8 hours of co-incubation. As expected, TNF pre-stimulation of endothelial cells was associated with a reduction in impedance within 24 hours (Figure 7B). Interestingly, the addition of mMP to TNF pre-treated endothelial cells also increased the impedance level before the TNF began to take effect, subsequently causing the impedance to gradually drop at the same rate as the control after approximately 24 hours.

In conjunction with the impedance assay, a permeability assay was performed. Endothelial cells, with or without TNF, were incubated either with non-stimulated or LPS-induced mMP before FITC-dextran 70 kDa was added. The 18 hour incubation of either non-stimulated or LPS-induced mMP with resting endothelial cells did not change the trans-monolayer passage of dextran suggesting no detrimental effect on endothelial monolayer integrity (Figure 7C). Similarly, pre-stimulation of the endothelial monolayer with “priming” doses of TNF (Figure 7D) did not change permeability to dextran. Treatment with TNF (100 ng/ml) induced a 25% increase in permeability, compared to an 80% change induced by the positive control cytochalasin D.

**Figure 8:**
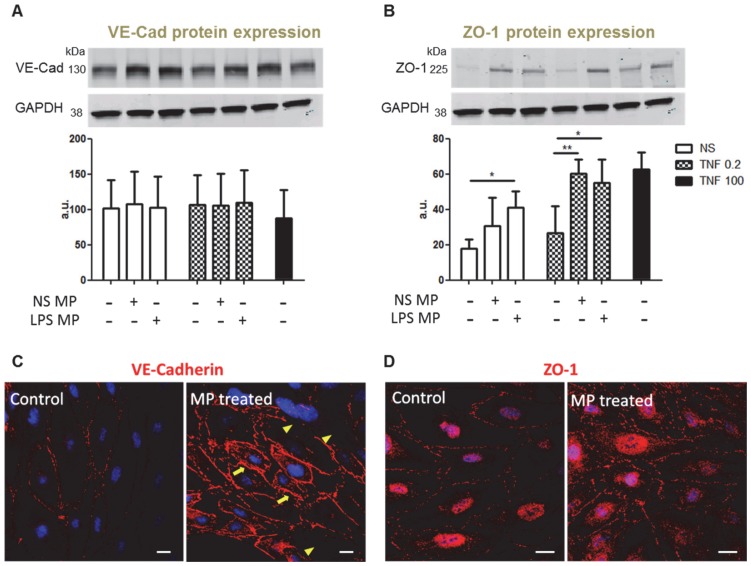
mMP modifies endothelial junctional protein expression. After overnight treatment of endothelial cells with mMP, no changes were observed in the levels of VE-Cadherin protein expression (A). Resting endothelial cells treated with mMP from resting monocytes (NS MP) and TNF primed endothelial cells treated with mMP from both resting and activated monocytes (LPS MP) displayed significantly higher levels of ZO-1 protein (B). Confocal microscopy revealed a redistribution of VE-Cadherin (C) and upregulation of cytosolic ZO-1 (D) upon mMP treatment. Data shown are representative of three independent experiments. Bar graphs are expressed as mean ± SD. Scale bars = 20 µm. *p<0.05, **p<0.01.

Finally, we investigated the potential modification of candidate junctional proteins by mMP. After co-culture with endothelial cells, Western blot analysis revealed that there were no significant changes in the translation of VE-Cadherin protein, whether or not endothelial cells were treated with mMP (Figure 8A). However, when the same cell lysates were probed for ZO-1, we observed an increased expression of ZO-1 protein in endothelial cells treated with mMP (Figure 8B). This was particularly significant in resting endothelial cells treated with mMP from LPS stimulated monocytes. Enhanced ZO-1 expression was also observed in TNF primed endothelial cells treated with mMP and independent of whether the mMP were derived from resting or activated monocytes.

Fluorescence microscopy revealed that confluent endothelial cells display smooth, continuous and homogenous junctional staining at the cell-cell contact when stained for VE-Cadherin in resting conditions (Figure 8C). Treatment with mMP, whether from resting or LPS-activated monocytes, showed increased staining in some areas (arrow) and almost no evident of junctional VE-Cadherin in others (arrow head), despite the endothelium remaining intact, suggesting a redistribution of the protein rather than a de novo synthesis. When observing ZO-1 expression, untreated endothelial displayed a low yet finely defined junctional staining pattern. Treatment with mMP mildly enhanced the staining in junctional areas but was noticeably stronger in the nuclear and cytosolic areas of the endothelial cells, which together with Western blot data, is suggestive of an increase in ZO-1 protein synthesis.

## Discussion

Current literature suggests that MP displaying a particular phenotype, whether pro- or anti-inflammatory, can transfer these properties onto their target cells. Based on the data generated in this work, we propose an alternate view to that of circulating MP exacerbating disease severity. Our goal was to investigate the properties of mMP and how they induce functional changes in brain microvascular endothelial cells in the context of inflammation and sepsis. By characterising the surface and mRNA profile of monocytic cell lines and their MP using flow cytometry and RT-qPCR, we built on this to decipher the functional outcome of the interactions with human brain microvascular endothelial cells using flow cytometry, confocal microscopy and trans-endothelial electrical resistance. This work addresses the vesiculation of these monocytes in relation to endothelial reactivity and demonstrates that mMP are inducing differential endothelial gene expression involved in a pathway considered anti-inflammatory rather than pro-inflammatory.

Numerous studies have showed that activation of cells instigates the release of MP [39], [40]. Our experimental data confirms that key stimulants such as TNF and LPS are capable of increasing release of MP from human brain endothelial and monocytic cell lines respectively. We then characterised the surface antigens and cytoplasmic content of mMP to determine whether they had similar or different properties from the activated mother cell. By studying a selection of molecules involved in adhesive, coagulatory and inflammatory processes capable of eliciting downstream endothelial cell dysfunction, we were able to extend on Bernimoulin *et al.*'s observation in the monoblastic THP1, that different stimuli could induce unique MP proteomic profiles [41]. A surface antigen phenotype comparison between THP1 and MM6 revealed a similar surface profile between the two cells lines. LPS treatment of MM6, maturer and phenotypically closer to circulating monocytes than the monoblastic THP1 [33], enhanced their expression pro-inflammatory surface markers such as CD80, CD86 and CD54 as well the expression of pro-inflammatory RNAs for IL-6, IL-8 and TNF. Examination of MP progeny from activated MM6 revealed a more pro-inflammatory profile that, with the exception of IL-6 and TLR4, mirrored their parent cells. These qualitatively different mMP thus carry potentially important biological properties. In the same way an activated monocyte profile can trigger activation of various target cells, the display of the very same markers by mMP equips them to also trigger downstream events caused by receptor-ligand interactions. Moreover, the bearing of mRNA by mMP suggests that after binding with their target cells, MP can act as intermediates of cell-cell communication and serve to amplify the effect caused solely by the parent cell [42]. Additionally, recent studies have also described that not only can MP transfer functional proteins [43], but that they are even able to convert proteins from the inert to its inflammatory form [44]. Furthermore, MP have also been described as a potential protective mechanism by which parent cells utilise against RNAse degradation to ensure the successful deliverance of intact microRNAs to target cells [45]. It is important to note that whilst it is possible that as transport vehicles, MP could also serve as a platform for further dissemination of endotoxin *in vivo*, the MP samples prepared in this *in vitro* study were free from detectable endotoxin demonstrating that the effects we observe are solely due to the MP and not due to the presence of LPS carried by the MP.

TF is well known to be highly expressed on the surface of activated monocytes and an important initiator of the coagulation cascade [9], [46], [47]. We found that TF was expressed on MM6 and was up-regulated by LPS stimulation (both surface protein and mRNA), however the corresponding MP showed little surface expression but did contain mRNA. We therefore aimed to assess whether TF and PS could synergistically increase the procoagulant potential of mMP derived from MM6. The effect on the clotting time was modestly enhanced by mMP derived from activated cells compared to resting cells, emphasising the importance of TF in coagulation. Our data shows that mMP are indeed procoagulant, however, this procoagulant potentials seems to be mainly TF-independent and more reliant on the presence of PS at the surface of the MP.

Of particular interest to this study, was the functional effect imparted by mMP onto endothelial cells. Various soluble agonists (including cytokines and other mediators) can augment cell vesiculation. Enhanced eMP production is known to be a hallmark of endothelial cell activation [39], [48] [49]. However, to our knowledge this study is the first to demonstrate that mMP themselves, can promote endothelial vesiculation. The higher numbers of eMP observed here in our *in vitro* model of brain inflammation was consistent with increased release of MP during inflammation observed in clinical studies [3]–[5]. Previous studies *in vivo* have also described worsening of pulmonary and capillary leak when treated with high numbers eMP [50]. The fact that LPS-induced mMP can activate endothelial cells and increase their eMP production to levels higher than those obtained with a maximal dose of TNF alone, provides further evidence that MP are not simply inert bystanders, but biologically active communicators that capable of modifying the response of their target cell. This is also supported by our data (not shown) and other's [51] showing that mMP can up-regulate adhesion molecules at the surface of endothelial cells.

We originally had hypothesised that interactions between MP produced by activated monocytes and endothelial cells would consequently result in endothelial cell dysfunction. However, in our experiments, while endothelial cells showed activation – as assessed by enhanced eMP release – under the influence of mMP, measurement of the endothelial impedance showed that these mMP may produce stabilization rather than breakdown of the endothelial monolayer. Previous work by Aharon *et al.* has demonstrated that ‘microvesicles’ consisting of MP together with exosomes, are capable of inducing endothelial apoptosis [52]. Such findings, together with our work, suggest that these different effects could be attributed to the differences between exosomes and MP – that they are not only distinguishable by size (40–100 nm vs. 0.1–1 µm) and origin (α-granule secreted vs. plasma membrane), but also by their effects on target cells. To our knowledge thus far, the only other MP capable of increasing impedance are those derived from platelets [53]. Monocytes on the other hand triggered a decrease of the TEER, suggesting a monolayer disruption and an opening of endothelial junctions. The tightening of cell-cell junctions observed here suggests that mMP, instead of amplifying the inflammatory response as expected, may be counteracting the deleterious effects of its mother cell to reduce the severity of endothelial injury. Both impedance and permeability results complementarily suggest that the mMP as applied here did not damage the endothelial cell monolayer.

In conjunction with the increase of impedance, mMP lowered the endothelial expression of activated Src without affecting levels of total Src. Src, a member of the non-receptor Src family tyrosine kinases is expressed in endothelial cells and regulates physiological functions such as cell adhesion, proliferation and migration [54]. Recent studies have found a strong correlation between the activation of Src and increased endothelium permeability [55], [56], whereby inhibition of Src prevented junctional protein phosphorylation and thus reduced permeability [57]. By modifying proteins involved in cell-cell junctions such as zonula occludens-1 and VE-cadherin, Src can cause gap formation leading to leaky vessels [58], [59]. In our case, mMP lowered the expression of activated Src, which seems consistent with the increase in impedance suggestive of a tightening of the monolayer. Such alteration of endothelial Src expression by mMP demonstrates that the aforementioned change in endothelial integrity is not solely the result of a direct contact but also of a signal transduction triggered within the endothelial cell. Aside from the Src modification demonstrated here, other studies have also shown that MP derived from LPS-treated monocytes can alter endothelial expression of signalling proteins such as ERK1/2 and NF-κB [51]. Src activation controls vascular permeability whereby a decrease of this activity by mMP is associated with reduced endothelium permeability.

Looking further downstream, we determined that mMP modification of endothelial permeability could indeed be attributed to the assembly or reorganisation of tight junctional proteins. Whilst no significant changes were observed in ZO-1 expression at the plasma membrane of cell-cell junction, treatment with mMP resulted in an accumulation of cytosolic ZO-1. Gilleron *et al.* suggest that the Src/ZO-1 relationship may be in part modulated by connexin 43, a transmembrane gap junction protein [60]. They report the recruitment of Src to the plasma membrane enhanced connexin 43/Src interactions whilst simultaneously driving the dissociation of connexin 43/ZO-1 complexes. Our work suggests that the mMP-induced diminishment of pSrc allows the retainment of ZO-1 localised at tight junctions whilst also prompting the protein synthesis of ZO-1. The contributory role of this enhanced cytosolic ZO-1 is still yet to be determined.

Previous studies have described Src inhibition leading to an impaired internalisation of VE-Cadherin and thus reduced permeability [57], [61]. Our data suggest that mMP prevent Src activation, and do not enhance VE-cadherin production. Rather, mMP trigger junctional protein redistribution with some areas of the endothelium displaying weaker VE-cadherin signals and others showing strong recruitment of VE-cadherin at cell peripheries to reinforce tight junctions, which, in part, could explain the observed reduction in permeability. Together, our data suggest that the Src regulated assembly and disassembly of tight junctions as reported by Dwyer *et al.* could be a pathway instigated by mMP [62].

In conclusion, this study is the first in the field of monocyte biology to indicate that mMP have a protective role despite being released by monocytes activated within a pathogenic environment. More broadly, aside from the traditional view of MP as amplifiers of the pro-inflammatory response, this study has found that LPS-induced mMP may actually display a dual potential by having a deleterious intrinsic phenotype but showing beneficial potential by preventing further inflammatory damage. Whether both potentials are active at the same time or sequentially and whether the protective or deleterious effect is dominant remains to be determined. Though further studies are required to appreciate where MP stand in the pathophysiology of septic shock, it is clear these circulating bioactive vesicles have contrasting effects in the intercellular communication network and in the subsequent protective function of the endothelium.

## Supporting Information

Figure S1 **Resting and LPS-stimulated THP1 were stained with anti-CD106, HLA-DR, CD80, CD86, CD11b, TF, CD14, CD31, CD54 mAb and annexin-V.** The mean fluorescence intensity was measured and compared to isotype-matched controls. Monocytes with MFI between 0–1, 1–5, and above 5 were considered as low expressors (top panel), medium expressors (middle panel) and high expressors (bottom panel) respectively. Experiments were performed three times in duplicates and expressed as mean ± SD. **p<0.01.
(TIF)

Figure S2 **Endothelial cells were TNF-primed or activated with high dose of TNF overnight and the levels of MP before treatment with mMP from either resting of LPS-stimulated THP1.** mMP did not significantly alter eMP release in resting endothelial cells. However, mMP derived from LPS-stimulated THP1 significantly enhanced eMP release from TNF-primed endothelium. Non-stimulated mMP did not induce any significant changes in TNF primed endothelial cells. Experiments were performed five times in duplicates or triplicates. Data are mean ± SD. **p<0.01.
(TIF)
