# Supplementary figures and images for: Endotoxin-Induced Monocytic Microparticles Have Contrasting Effects on Endothelial Inflammatory Responses
Source: PLoS One. 2014 Mar 19;9(3):e91597. doi: 10.1371/journal.pone.0091597 (PMC3960107; doi:10.1371/journal.pone.0091597)

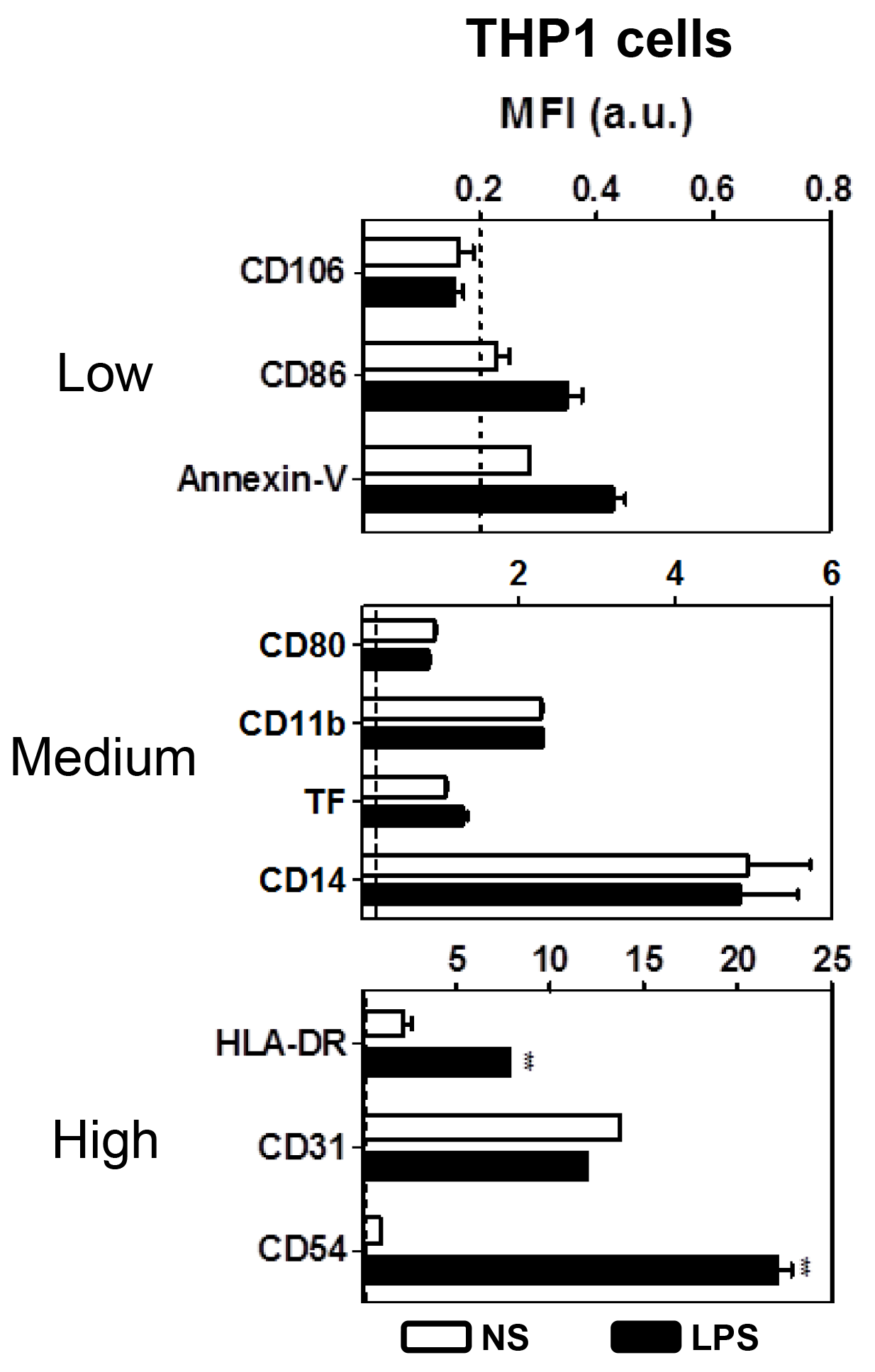

Supplement: Figure S1 — Resting and LPS-stimulated THP1 were stained with anti-CD106, HLA-DR, CD80, CD86, CD11b, TF, CD14, CD31, CD54 mAb and annexin-V. The mean fluorescence intensity was measured and compared to isotype-matched controls. Monocytes with MFI between 0–1, 1–5, and above 5 were considered as low expressors (top panel), medium expressors (middle panel) and high expressors (bottom panel) respectively. Experiments were performed three times in duplicates and expressed as mean ± SD. **p<0.01. (TIF) [file pone.0091597.s001.tif]

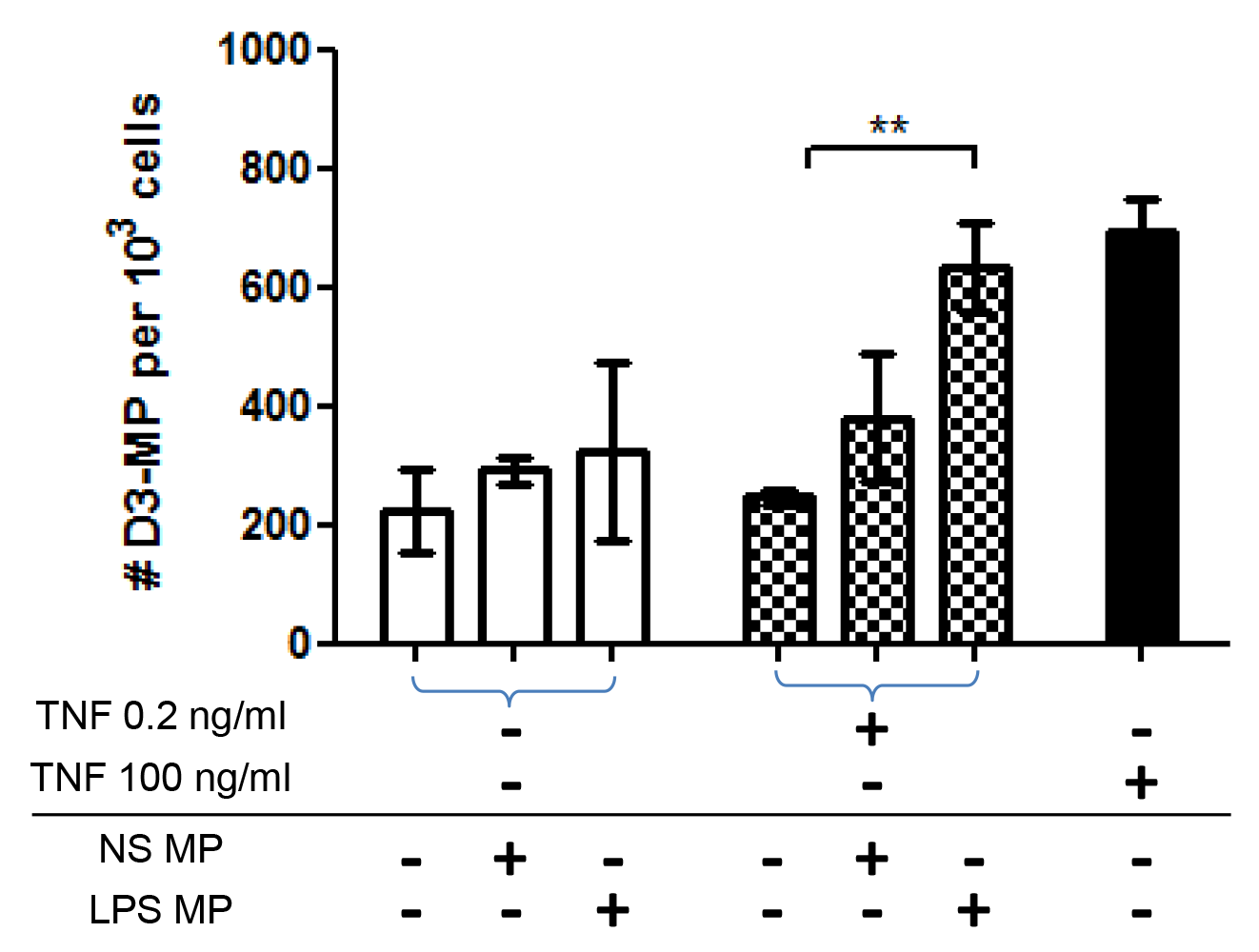

Supplement: Figure S2 — Endothelial cells were TNF-primed or activated with high dose of TNF overnight and the levels of MP before treatment with mMP from either resting of LPS-stimulated THP1. mMP did not significantly alter eMP release in resting endothelial cells. However, mMP derived from LPS-stimulated THP1 significantly enhanced eMP release from TNF-primed endothelium. Non-stimulated mMP did not induce any significant changes in TNF primed endothelial cells. Experiments were performed five times in duplicates or triplicates. Data are mean ± SD. **p<0.01. (TIF) [file pone.0091597.s002.tif]
